# Long Term Outcomes in Stress Echocardiography: Ten Year Follow up of a Cohort in a Single Centre

**DOI:** 10.4021/cr133w

**Published:** 2012-01-20

**Authors:** Reza Ashrafi, Ewan McKay, Julia Jones, Aham Amadi

**Affiliations:** a University Hospital Aintree NHS Foundation Trust, University Hospital Aintree, Lower Lane, Liverpool L9 7AL, UK; b Royal Oldham Hospital NHS, Pennine Acute NHS Trust, Rochdale Road, Oldham, Manchester OL1 2JH, UK

**Keywords:** Stress echocardiography, Outcomes, Angiography

## Abstract

**Backgroud:**

The high service burden for acute admissions and referrals via rapid access chest pain clinics for evaluation of possible coronary artery disease means that many patients are now undergoing an investigation such as stress echocardiography as part of their evaluation. We aimed to see if the reassurance provided by negative stress echocardiography correlates with long-term event free survival.

**Methods:**

A cohort of all patients who were referred at a single centre for stress echocardiography for diagnosis of coronary artery disease between January 1st 1999 and December 31st 2000 were followed up at least 10 years following theirs stress echocardiogram for further major cardiovascular events and mortality.

**Results:**

A total of 64 patients were identified where records could be obtained for analysis. There were 16 positive scans, 37 negative scans and 11 inconclusive scans. The indeterminacy rate of scans was 17%, the sensitivity rate for detecting significant disease as indexed to invasive angiography was 88 % and the specificity rate compared with angiography was 75%. There were no myocardial infarctions or new diagnoses of heart failure in the negative echocardiogram group. There were seven deaths in the total population and only one death from cardiovascular causes in the negative echocardiogram group.

**Conclusion:**

Stress echocardiography even in this small group predicts long-term outcomes as well as invasive coronary angiography.

## Introduction

Stress echocardiography was initially developed approximately 20 years after the initial introduction of cardiac ultrasound in 1979 and was limited by poor technical apparatus [1].

Since then there has been rapid development in terms of techniques, indications and the accuracy of the test with thousands of people undergoing stress echocardiography each year. This rapid expansion in testing has rested on numerous works showing that stress echocardiography has evidence in predicting cardiovascular outcomes [2].

More specifically what has been shown in a meta-analysis of available work with a follow up period of approximately 33 months is that a negative stress echocardiogram, very strongly confers a low likelihood of future major adverse cardiovascular events [3]. There have been very few studies looking at the number of events in the longer term [4] and they have not previously been over as long a period (10 years or more) as the traditional gold standard investigation, invasive coronary angiography [5].

### Reasons for this study

The role of imaging in cardiology is increasing year on year and the current recommendations from the European society of Cardiology suggest usages for echocardiography which covers a very large proportion of patients [6].

In the United Kingdom (UK), the recently published guidance from the national institute for clinical excellence (NICE) has clarified the role for stress echocardiography in the assessment of people with stable chest pain [7]. As stress echocardiography is recommended for the assessment those with a moderate pretest likelihood of coronary artery disease, it is likely the number of stress echocardiograms performed will increase.

Most hospitals in the United Kingdom are unlikely to have access to cardiac magnetic resonance imaging or myocardial perfusion single photon emission computed tomography, which are the two other recommended options in the moderate risk group. We therefore felt that as our centre is a non-specialist centre as our the majority of centers in the UK, we needed to clarify how useful a negative a result would be for the people seen and assessed by stress echocardiography.

What we aimed to establish with this audit was whether the strong negative predictive value of stress echocardiography for future events in the short term was replicated in the long term.

## Methods

### Patient selection

The patient list was formed from a list of patients kept by the department of cardiology who underwent stress echocardiography between January 1st 1999 and December 31st 2000. All patients were referred for stress echocardiography from within Aintree University Hospitals NHS Foundation Trust to a single operator. All patients who were referred for the assessment of possible ischaemic heart disease were included, patients with known disease undergoing viability assessments or those with valvular pathology being further assessed were not included in the analysis.

### Ethical approval

As part of the ongoing service evaluation and audit process, it was not felt that this work required referral to the national ethics system. This work was however formally approved by the audit department university hospital Aintree NHS foundation trust.

### Stress echocardiography protocol

Patients were admitted to the department following the omission of all rate limiting drugs or caffeine containing products for 48 hours. Patients were put on continuous electrocardiographic monitoring and regular blood pressure monitoring. Patients were given a pharmacological stressor either arbutamine [8] or dobutamine [9] in standard protocols described elsewhere. Patients were then lied in the left lateral position and underwent standard two dimensional imaging using a HP Sonos 1000 echocardiography system (Hewlett-Packard, Callifornia, USA) by a British society of echocardiography accredited sonongrapher. In some cases Optison contrast agent (General Electric, USA) was used to increase the endocardial border definition for analysis as described in other papers [10].

An examination was considered to have reached the required heart rate for examination at the discretion of the operator but normally a level of at least 85% of age related maximum was used. The images were then analysed offline by a single experienced operator (AA-consultant cardiologist). Examinations were considered diagnostic of ischaemia and therefore positive when 2 or more adjacent regions became hypokinetic with stress in line with published guidelines [11].

### Result analysis

Results were collated in terms of the age, sex and risk factor distribution of the patients scanned. We then collated the number of positive, negative and inconclusive echocardiograms recorded and then the sensitivity and specificity of the test by comparing the echocardiograms where there was an invasive coronary angiogram after and if that showed significant flow limiting disease. We also made a record of the percentage of age predicted maximum heart rate that was achieved where that information was available.

We then collated how many patients went on to develop myocardial infarctions, heart failure and who died from all cause and cardiovascular causes (myocardial infarctions, heart failure and arhythmogenic death). Follow up was performed using the patient’s casenotes, electronic data and if needed by correlation with the general practitioner’ s records.

## Results

In all we found 64 patients who had stress echocardiography performed in the period from the 1st of January 1999 to the 31st of December 2000 for the attempted primary diagnosis of myocardial ischaemia.

The average age of patients presenting was 56 (95% CI 54 to 58) with fifty one percent being female.

The average heart rate as a percentage of the age predicted maximum reached for all stress echocardiograms performed was 83% (95% CI 81 to 85).

The risk factor profile of the patients involved in the study is shown below in Table 1.

**Table 1:** Patient Risk Factors

Risk Factor	Percentage of Patients
Diabetes Mellitus	11
Smoker	23
Hypertension	46
Cholesterol	40
Family History of Coronary artery disease	28

### Echocardiogram analysis

In our cohort of 64 patients there were16 positive scans, 37 negative scans and 11 inconclusive scans.

The indeterminacy rate in our collection of echocardiograms was 17% and the sensitivity of stress echocardiography in our cohort was 88% and the specificity 75% using the standard method.

### Outcomes

The primary outcome measurements were in all cause and cardiovascular mortality and major adverse cardiovascular events (myocardial infarction and development of heart failure).

In all there 7 deaths, 2 in the positive echocardiogram group from cardiovascular causes, 1 in the inconclusive echocardiogram group from cardiovascular causes and four in the negative echocardiogram group.

In this negative echocardiogram group there were 2 deaths not related to cardiovascular causes and 1 death from cardiovascular causes (witnessed ventricular fibrillation- with no obtainable post mortem report). The cause for one patient could not be determined due to a lack of correct address and general practitioner details.

This equates over the minimum ten year follow up to a 0.27% per year risk of death from a cardiovascular cause following a negative stress echocardiogram.

The results for the patients developing major adverse events such as heart failure and myocardial infarction are shown below (Table 2).

**Table 2:** Major Events by Echocardiogram Result

Echocardiogram Result	Myocardial Infarction	Heart Failure
Positive	2	3
Negative	0	0
Inconclusive	2	2

### Statistical analysis

Using a Mid P exact test, there was no significant difference between the all cause and cardiovascular mortality rates in the positive and negative echocardiogram groups, owing to the low numbers involved.

Using a Yates corrected Chi-Squared test there was a significant difference between myocardial infarction and rates of heart failure between the positive stress echocardiograms and the negative stress echocardiogram (P < 0.001).

## Discussion

What this studies expands on compared to previous work is that high reassurance provided by a stress echocardiogram in the short term [12] is replicated in the longer term, in terms of major cardiovascular events (P < 0.001) and mortality rates (statistically non significant).

When compared to a negative invasive coronary angiogram the per year risk of cardiovascular death rate of 0.51% [5] is very similar to the same rate for a negative stress echocardiogram found here, 0.27%.

### Limitations

Our sample is a small sample with a single operator and as such is open to biases associated with smaller studies. We also assumed invasive coronary angiography to be the gold standard and that luminographical analysis when negative excluded significant disease. There is more and more work using intravascular ultrasound that suggests that unstable plaques and varying plaque morphology may be more significant than operators may appreciate with traditional angiography [13]. This may mean that ischaemia identified on stress echocardiography but not on angiography may not be a false negative just a limitation of standard angiography. Our cohorts were all tested using pharmacological stressors and it maybe that the more physiological stress produced with exercise may have yielded different results.

The final limitation to our study is that because all the echocardiograms were analysed by one highly experienced reader, there is an inherent expert bias in terms of the accuracy of the results. It maybe that when replicated with less experienced readers that the interpretation of the echocardiograms may not be as accurate and the outcomes not as favourable.

### Implications

As our results fit in broadly with in terms of sensitivity, specificity and previously reported mortality rate and adverse event rates published in the shorter follow up period for new referrals [14], we feel that our data though small is likely to be representative in a larger population. This implies that the likely expansion in functional imaging which in many trusts such as our own is likely to be via stress echocardiography is likely to prove safe in the long term.

As ever cost effectiveness is a major aspect of all work performed in the national health service and stress echocardiography has been shown to a very cost effective way of reducing the need for invasive angiography [15] and now it is clear that money saved is not likely to be wasted when there are late events in follow up.

### Future directions

It would of course be interesting to repeat this work in a larger group with more operators to see if our results are replicated. With improvements in the scanner technology behind stress echocardiography, we expect the accuracy and the predictive value to improve. We suggest also that correlation between positive echocardiograms and apparently normal angiography be repeated with intravascular ultrasound or optical coherence tomography to more accurately visualise vessels and plaque.

### Conclusions

Negative stress echocardiography is as good a negative predictor of cardiovascular mortality.

## Key Messages

1) Negative Stress Echocardiography predicts long term freedom from major cardiovascular events; 2) Mortality rates following a negative stress echocardiogram are similar to a negative coronary angiogram; 3) Stress Ehocardiography is cost effective in the long term as savings identified in the short term are not affected by a high late event rate.
